# Serum interleukin-17 predicts severity and prognosis in patients with community acquired pneumonia: a prospective cohort study

**DOI:** 10.1186/s12890-021-01770-6

**Published:** 2021-12-02

**Authors:** Chun-Mei Feng, Xin-Ming Wang, Meng-Die Li, Zheng Xu, Dong-Xu Hua, Jia-Yi Cheng, Ling Zheng, Hui Zhao, Lin Fu

**Affiliations:** 1 Respiratory and Critical Care Medicine, Second Affiliated Hospital of Anhui Medical University, Furong Road No 678, Hefei, 230601 Anhui China; 2 Department of Pharmacy, First Affiliated Hospital of Anhui Medical University, Hefei, 230022 Anhui China

**Keywords:** Community-acquired pneumonia, Interleukin-17, CAP severity scores, Prognosis, Biomarker

## Abstract

**Background:**

Some studies previously demonstrated that interleukin-17 (IL-17) involves in pulmonary diseases progression. Nevertheless, the role of IL-17 in community-acquired pneumonia (CAP) remains unknown. This study aims to examine the correlations between serum IL-17 with the severity and prognosis in CAP patients through a prospective cohort study.

**Methods:**

All 239 CAP patients were recruited. Serum IL-17 was detected by enzyme-linked immunosorbent assay (ELISA). The CAP severity was evaluated through CAP severity scores, including CURB-65, CRB-65, PSI, SMART-COP, CURXO and APACHE II.

**Results:**

Serum IL-17 was gradually increased consistent with the severity of CAP. Correlative analysis suggested that serum IL-17 was associated with clinical physiologic indicators among CAP patients. Logistic regression indicated that serum IL-17 was positively related to CAP severity scores. Additionally, the prognostic outcomes were tracked among CAP patients. The levels of IL-17 on admission were significantly increased in CAP patients with ICU admission, mechanical ventilation, vasoactive agent, death and longer hospitalization days. Logistic regression analyses revealed serum higher IL-17 on admission elevated the risks of vasoactive agent usage and longer hospital stays in CAP patients. The cut-off concentrations of serum IL-17 for death, ICU admission, mechanical ventilation and ≥ 14 hospital stays were 86.80 ng/mL, 84.92 ng/mL, 84.92 ng/mL and 60.29 ng/mL respectively.

**Conclusions:**

Serum IL-17 on admission is positively associated with the severity and poor prognosis among CAP patients, revealing that IL-17 may implicate in the pathological process of CAP. Therefore, serum IL-17 may become an effective biomarker for diagnosis, prognosis and therapy for CAP patients.

## Introduction

Community-acquired pneumonia is a lung infection as a result of a wide variety of microorganisms, mainly including bacterial pathogens (such as *Streptococcus pneumoniae*, *Mycoplasma pneumoniae* and *Staphylococcus aureus,* et al.) and viral (such as human rhinovirus, influenza, parainfluenza virus, respiratory syncytial virus, et al.) [1]. CAP always causes immune system disorders, local and systemic higher inflammatory responses in patients [2]. CAP is consistently a leading cause of respiratory illness around the world [3, 4]. It disproportionately affects adults older than 60 years old or children younger than five years old. CAP has resulted in over 60,000 deaths annually and more than 10 billion dollars health care costs in United States [4–7]. Patients with mild infections are less likely to seek medical attention and diagnosis. Therefore, the true incidence of CAP may be underestimated [8]. If the diagnosis and treatment were not conducted timely and properly, the rate of fatality will rise sharply [9]. Although validated pneumonia severity scores can guide the decision between outpatient and inpatient therapy, appropriate indicators as biomarkers for CAP may further assist with risk stratification. Therefore, a typical biomarker is imminently explored to improve early clinical diagnosis and decrease the complications. As we know, early detection of CAP is critical in improving prognosis for CAP patients.

Interleukin-17 (IL-17) is primarily secreted by T helper 17 cells (Th17), monocyte and eosinophilia [10, 11]. Increasing data have proved that IL-17 can recruit neutrophils, activate T cells, stimulate macrophages and epitheliums. Finally, IL-17 produces a range of pro-inflammatory cytokines and induces inflammatory reaction in the bodies [12, 13]. Past works have indicated that IL-17 is highly expressed in rheumatoid arthritis and ulcerative colitis [14, 15]. IL-17 also has been shown to play central roles in the process of several lung diseases, including lung fibrosis, emphysema, acute lung injury and pulmonary hypertension [16–19]. Moreover, in vitro research has confirmed that IL-17 overexpression or recombinant IL-17 administration elevates chemokines and evokes inflammatory reaction of lung [20, 21].

At present, IL-17 is considered to an attractive target for inflammatory responses in the bodies. However, the physiological function of IL-17 was not obvious in CAP patients at present. The associations of IL-17 with severity and clinical outcomes of CAP patients were unclear. We speculate that IL-17 takes part in the pathophysiology process of CAP. Consequently, the study aims to explore the associations of serum IL-17 with the severity and prognosis in CAP patients through a prospective cohort study.

## Methods

### Study design and subjects

All CAP patients were enrolled from August 2019 to April 2021 in the Department of Respiratory and Critical Care Medicine of Second Affiliated Hospital. At first, all 288 patients were recruited and agreed to take participate in this research. Twenty-eight patients with incomplete information, 12 lost cases and 9 withdrawing cases were excluded from this research. Finally, 239 CAP patients were enrolled in the present research. All CAP patients were eligible for this research. After obtaining informed consent from the patients, we gathered relevant demographic characteristics and clinical information. At the onset of CAP, CAP patients always showed specific symptoms and signs, primarily including fever, cough, expectoration, and even purulent sputum; with or without chest pain; chest tightness and dyspnea in CAP patients with affected multiple pulmonary lobes. All selected 239 CAP patients in this research must meet certain diagnostic standard: In accord with the clinical manifestations of pneumonia and consistent with chest CT scans, mainly including (1) chest radiograph suggesting either a new patchy infiltrate, leaf or segment consolidation, ground glass opacity or interstitial change; (2) at least one of the following signs: the presence of cough, sputum production and dyspnoea; core body temperature higher than 38.0 °C; auscultatory findings of abnormal breath sounds and rales; or the counts of white blood cell more than 10 × 10^9^ L or less than 4 × 10^9^ L; (3) occurred in the community, rather than in a hospital [22]. The inclusion criteria were as follows: pneumonia was occurred in the community or within a definite incubation period after hospitalization; these candidates were not admitted to the hospital for the last three months; not pregnant; no other pulmonary diseases, such as pulmonary malignant tumor, pulmonary tuberculosis or immunodeficiency [23, 24]; no antibiotic treatment or intervention before hospitalization. Peripheral blood samples were collected and anticoagulated with EDTA within 24 h after hospitalization. Blood routine examination and biochemical detection were performed in all CAP patients. Then, the severity of pneumonia was evaluated using well-recognized CAP severity scores, including Pneumonia Severity Index (PSI), CURB-65, CRB-65, SMART-COP and Acute Physiology and Chronic Health Evaluation II (APACHE II). Moreover, CAP patients were divided into mild patients and severe patients through CURXO score. Severe CAP is a group of patients who requires treatment in the intensive care unit (ICU). Our research has obtained approval from Ethics Committee of Second Affiliated Hospital of Anhui Medical University (YX2021-085). Furthermore, written agreement consent from eligible patients or the patient’s next of kin were obtained.

### Enzyme-linked immunosorbent assay (ELISA)

Before any treatment, peripheral blood samples were collected and centrifuged. Then samples were stocked in the − 87 °C ultracold refrigerator until use. IL-17 ELISA kits (CSB-E12819h) were purchased from Cusabio, Wuhan, China (https://www.cusabio.com/). IL-6 ELISA kits (JYM1942Hu) were obtained from Wuhan Colorful Gene Biological Technology Co. The concentrations of serum inflammatory cytokines were detected following the instructions closely [24, 25].

### Statistical analysis

All statistical analyses were conducted using SPSS 19.0 version software. Demographic characteristics and laboratory parameters were shown as mean ± standard error (SEM) or median with interquartile ranges. Categorical variables were expressed with frequencies and percentages. The correlations between serum IL-17 and clinical characteristics were evaluated using Pearson correlation analysis among CAP patients. The association between serum IL-17 with CAP severity scores and prognostic outcomes were accessed using logistic regression analysis. There were no significant differences (*P* > 0.05).

## Results

### Demographics characteristics and clinical information

In this study, 239 CAP patients were recruited. Demographic characteristics and laboratory test results were collected and generalized. The average age was 61.09 years old among CAP patients (Table 1). Among them, female patients accounted for 40.17%. The proportion of smoking in CAP patients was 17.57%. Besides, the comorbidities of CAP patients were analyzed. As shown in Table 1, 64 (26.78%) patients were with hypertension, 22 (9.21%) patients were with diabetes mellitus, 20 (8.37%) patients were with cerebral infarction, 11 (4.60%) patients were with coronary heart disease, 19 (7.95%) patients were with bronchitis and 78 (32.64%) patients were with other diseases. Blood cell content indicated that the count of white blood cell (WBC) was 8.26 × 10^9^/L, the count of neutrophil was 7.79 × 10^9^/L, the count of lymphocyte was 2.00 × 10^9^/L (Table 1). Meanwhile, liver function, renal function and myocardial function were tested among CAP patients. In addition, the median hospital stay is 10.00 days among CAP patients. During hospitalization, 71 cases admitted to the ICU, median hospital stays were 3.21 days, 66 cases were conducted with mechanical ventilation, 34 cases were used with vasoactive agent and 22 cases were died among CAP patients (Table 1). According to CURXO score, CAP patients who met the following two or more criterions were regraded as severe cases: confusion; urea nitrogen > 30 mg/L; respiratory rate > 30/min; abnormal shadows in multilobar or bilateral lungs; PaO_2_ < 54 or PaO_2/_FiO_2_ < 250 mmHg. Severe CAP patients accounted for 27.62% in CAP patients. The mean of CURB-65, CRB-65, PSI, SMART-COP and APACHE II was 1.31, 1.07, 78.72, 1.97 and 7.95, respectively.

**Table 1:** Demographic characteristics of participators at baseline

Variables	CAP (n = 239)
Age (years)	61.09 ± 1.14
Female, n (%)	96 (40.17)
BMI	22.45 ± 0.33
Smoking, n (%)	42 (17.57)
ICU admission, n (%)	71 (29.71)
ICU stay (days)	3.21 ± 0.65
*Comorbidities*	
Hypertension, n (%)	64 (26.78)
Diabetes mellitus, n (%)	22 (9.21)
Cerebral infarction, n (%)	20 (8.37)
Coronary heart disease, n (%)	11 (4.60)
Bronchitis, n (%)	19 (7.95)
Other disease, n (%)	78 (32.64)
*Blood routine*	
White blood cell (10^9^/L)	8.26 ± 0.27
Neutrophil (10^9^/L)	7.79 ± 0.75
Lymphocyte (10^9^/L)	2.00 ± 0.40
Monocyte (10^9^/L)	0.60 ± 0.04
Eosinophil (10^9^/L)	0.11 ± 0.01
Basophil (10^9^/L)	0.16 ± 0.07
*Liver function*	
Alanine aminotransferase (U/L)	23.00 (15.00, 42.00)
Alanine transaminase (U/L)	25.50 (18.00, 39.50)
*Renal function*	
Urea nitrogen (mmol/L)	5.25 (3.74, 7.20)
Creatinine (μmol/L)	63.00 (48.00, 83.25)
Uric acid (μmol/L)	259.50 (194.50, 347.25)
*Myocardial function*	
Creatine kinase (U/L)	68.00 (37.00, 114.00)
Creatine kinase isoenzyme (U/L)	13.00 (8.00, 20.00)
Lactate dehydrogenase (U/L)	211.00 (157.00, 294.00)
Cardiac troponin I (ng/mL)	0.02 (0.01, 0.05)
Myoglobin (ng/mL)	43.20 (28.25, 96.50)
*CURB-65, n (%)*	
0–1	148 (61.93)
2	50 (20.92)
3–5	41(17.16)
*CRB-65, n (%)*	
0	84 (35.15)
1–2	128 (53.56)
≥ 3	27 (11.30)
*PSI, n (%)*	
I (< 50)	54 (22.59)
II (51–70)	61 (25.52)
III(71–90)	55 (23.01)
IV (91–130)	48 (20.08)
V (> 130)	21 (8.79)
*CURXO, n (%)*	
Mild	173 (72.39)
Severe	66 (27.62)
*SMART-COP, n (%)*	
0–2	174 (72.80)
3–4	18 (7.53)
5–6	33 (13.81)
7–8	14 (5.86)
*APACHE * II* , n (%)*	
≤ 4	84 (35.15)
4–6	52 (21.75)
6–10	53 (22.18)
≥ 10	50 (20.92)

BMI, Body Mass Index; ICU, intensive care unit; PSI, Pneumonia Severity Index; APACHE II, acute physiology and chronic health evaluation II; CURB-65, acronym for confusion, urea, respiratory rate, blood pressure and age ≥ 65; CRB-65, acronym for confusion, respiratory rate, blood pressure and age ≥ 65; CURXO, acronym for confusion, urea, respiratory rate, X-ray and oxygen; SMART-COP, acronym for systolic blood pressure, multilobar infiltrates, albumin, respiratory rate, tachycardia, confusion, oxygen and pH

### The level of serum IL-17 in CAP patients

**Fig. 1:**
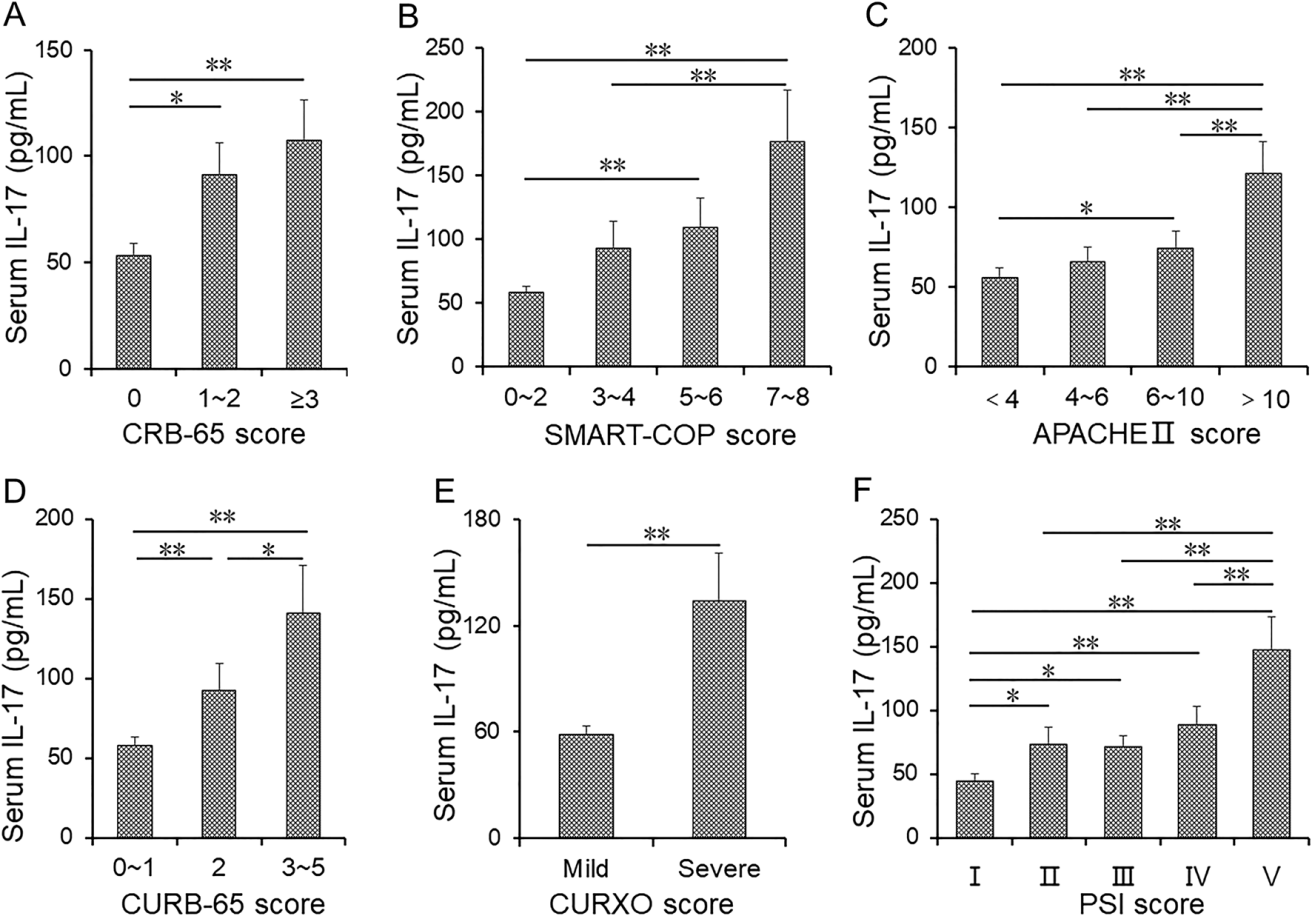
The levels of serum IL-17 in CAP patients with different severity scores. **A**–**F** The levels of serum IL-17 in CAP patients with different severity scores. **A** CRB-65 score. **B** SMART-COP score. **C** APACHE II score. **D** CURB-65 score. **E** CURXO score. **F** PSI score. All data were expressed as mean ± SEM. *P < 0.05, **P < 0.01

The concentration of serum IL-17 was measured and compared among various groups of CAP patients. The severity degrees of pneumonia were assessed by using CAP scoring system, such as CURXO, PSI, CURB-65, CRB-65, SMART-COP and APACHE II. Serum IL-17 was decreased in 0 score than those in the groups of 1–2 and ≥ 3 scores based on CRB-65 score (Fig. 1A). In accordance with SMART-COP score, serum IL-17 was lower in the group of 0–2 and 3–4 scores than those in the group of 7–8 score (Fig. 1B). IL-17 was higher in the group of 5–6 score than these in the group of 0–2 score (Fig. 1B). According to APACHE II score, IL-17 was highest in the group of > 10 score than other groups (Fig. 1C). The level of IL-17 was elevated in the group of 6 ~ 10 score compared with the group of < 4 score (Fig. 1C). Based on CURB-65 score, IL-17 was gradually increased in line with CURB-65 score (Fig. 1D). Additionally, IL-17 was lower in severe group than these in mild group (CURXO score) (Fig. 1E). Based on PSI score, serum IL-17 was gradually elevated with PSI score elevation (Fig. 1F).

### Associations of serum IL-17 with clinical characteristics among CAP patients

The correlations between serum IL-17 and blood routine indices were analyzed among CAP patients. As shown in Table 2, serum IL-17 was positively correlated with WBC (*r* = 0.28, *P* < 0.01) and neutrophil (*r* = 0.31, *P* < 0.01), inversely associated with lymphocyte (*r* = − 0.13, *P* = 0.02) and eosinophil (*r* = − 0.12, *P* = 0.04) in CAP patients. Moreover, the relationships of IL-17 with renal function, liver function and myocardial function were evaluated among CAP patients. The results revealed that IL-17 was positively linked with uric acid (*r* = 0.16, *P* < 0.01), urea nitrogen (*r* = 0.13, *P* = 0.03), alanine aminotransferase (ALT) (*r* = 0.19, *P* = 0.02), alanine transaminase (AST) (*r* = 0.25, *P* < 0.01) and creatine kinase isoenzyme (CKMB) (*r* = 0.16, *P* = 0.03) in CAP patients (Table 2). In addition, we found that there were positive correlations between serum IL-17 with platelet count (PLT) (*r* = 0.13, *P* = 0.03), d-dimer (*r* = 0.22, *P* < 0.01), B-type natriuretic peptide (BNP) (*r* = 0.21, *P* = 0.01) and fibrinogen (FIB) (*r* = 0.15, *P* = 0.02) among CAP patients. Finally, the associations between serum IL-17 and inflammatory cytokines were accessed. As shown in Table 2, the results indicated that serum IL-17 was positively and obviously correlated with C-reactive protein (CRP) (*r* = 0.36, *P* < 0.01) and interleukin-6 (IL-6) (*r* = 0.37, *P* = 0.02).

**Table 2:** Associations between serum IL-17 and clinical characteristics in CAP patients

Variables	WBC	Neutrophil	Lymphocyte	Monocytes	Eosinophil	Basophil
*r*	0.28	0.31	− 0.13	0.06	− 0.12	0.02
*P*	< 0.01	< 0.01	0.02	0.20	0.04	0.39

Variables	Uric acid	Urea nitrogen	Creatinine	ALT	AST	CK
*r*	0.16	0.13	0.07	0.19	0.25	− 0.03
*P*	< 0.01	0.03	0.17	0.02	< 0.01	0.36

Variables	CKMB	LDH	α-HBDE	D-dimer	Prothrombin	BNP
*r*	0.16	0.12	0.06	0.22	0.04	0.21
*P*	0.03	0.06	0.24	< 0.01	0.27	0.01

Variables	PCT	FIB	Hb	PLT	IL-6	CRP
*r*	0.27	0.15	− 0.02	0.13	0.37	0.36
*P*	< 0.01	0.02	0.36	0.03	0.02	< 0.01

### Associations of serum IL-17 with CAP severity scores in CAP patients

The concentration of serum IL-17 was divided into lower than 75% quartile and higher than 75% quartile. Then, the associations were accessed between serum IL-17 and CAP severity scores through logistic regression analysis in CAP patients. Univariable logistic regression analysis found that serum IL-17 was positively associated with 3–4 score (OR = 9.16; 95% CI 3.16, 26.57), 5–6 score (OR = 4.39; 95% CI 1.90, 10.12) and 7 ~ 8 score (OR = 23.50; 95% CI 6.07, 90.95) of SMART-COP, > 10 score of APACHE II (OR = 5.30; 95% CI 2.32, 12.09), ≥ 3 score of CRB-65 (OR = 4.79; 95% CI 1.83, 12.51), 2 score (OR = 3.37; 95% CI 1.58, 7.21) and 3–5 score (OR = 5.38; 95% CI 2.47, 11.75) of CURB-65, IV grade (OR = 3.11; 95% CI 1.08, 8.96) and V grade (OR = 22.00; 95% CI 5.86, 82.65) of PSI, severe patients of CURXO (OR = 5.98; 95% CI 3.10, 11.53) (Table 3). After age and sex were adjusted, further multivariate regression analysis revealed that IL-17 was positively associated with SMART-COP, APACHE II, CRB-65, CURB-65, PSI and CURXO in CAP patients (Table 3).

**Table 3:** Associations between serum IL-17 and CAP severity scores in CAP patients

Variables	Univariable, OR (95% CI)	*P*	Multivariable^a^, OR (95% CI)	*P*
*SMART-COP*				
0–1	1	–	1	–
3–4	9.16 (3.16, 26.57)	< 0.01	8.93 (3.00, 26.55)	< 0.01
5–6	4.39 (1.90, 10.12)	< 0.01	3.63 (1.53, 8.62)	< 0.01
7–8	23.50 (6.07, 90.95)	< 0.01	18.12 (4.50, 72.99)	< 0.01
*APACHE * II				
< 4	1	–	1	–
4–6	0.95 (0.36, 2.47)	0.91	0.84 (0.30, 2.37)	0.74
6–10	1.47 (0.60, 3.60)	0.40	1.26 (0.47, 3.39)	0.64
> 10	5.30 (2.32, 12.09)	< 0.01	4.03 (1.57, 10.30)	< 0.01
*CRB-65*				
0	1	–	1	–
1–2	1.74 (0.84, 3.58)	0.14	1.29 (0.57, 2.91)	0.55
≥ 3	4.79 (1.83, 12.51)	< 0.01	3.32 (1.18, 9.35)	0.02
*CURB-65*				
0–1	1	–	1	–
2	3.37(1.58, 7.21)	< 0.01	2.80 (1.27, 6.18)	0.01
3 ~ 5	5.38 (2.47, 11.75)	< 0.01	4.11 (1.78, 9.53)	< 0.01
*PSI*				
I	1	–	1	–
II	1.75 (0.60, 5.15)	0.31	1.50 (0.46, 4.90)	0.50
III	1.75 (0.58, 5.23)	0.32	1.36 (0.38, 3.93)	0.64
IV	3.11 (1.08, 8.96)	0.04	2.40 (0.67, 8.67)	0.18
V	22.00 (5.86, 82.65)	< 0.01	15.41 (3.34, 70.03)	< 0.01
*CURXO*				
Mild	1	–	1	–
Severe	5.98 (3.10, 11.53)	< 0.01	5.01 (2.55, 9.86)	< 0.01

^a^Adjusted for age and sex

### The levels of serum IL-17 in CAP patients with different prognostic outcomes

**Fig. 2:**
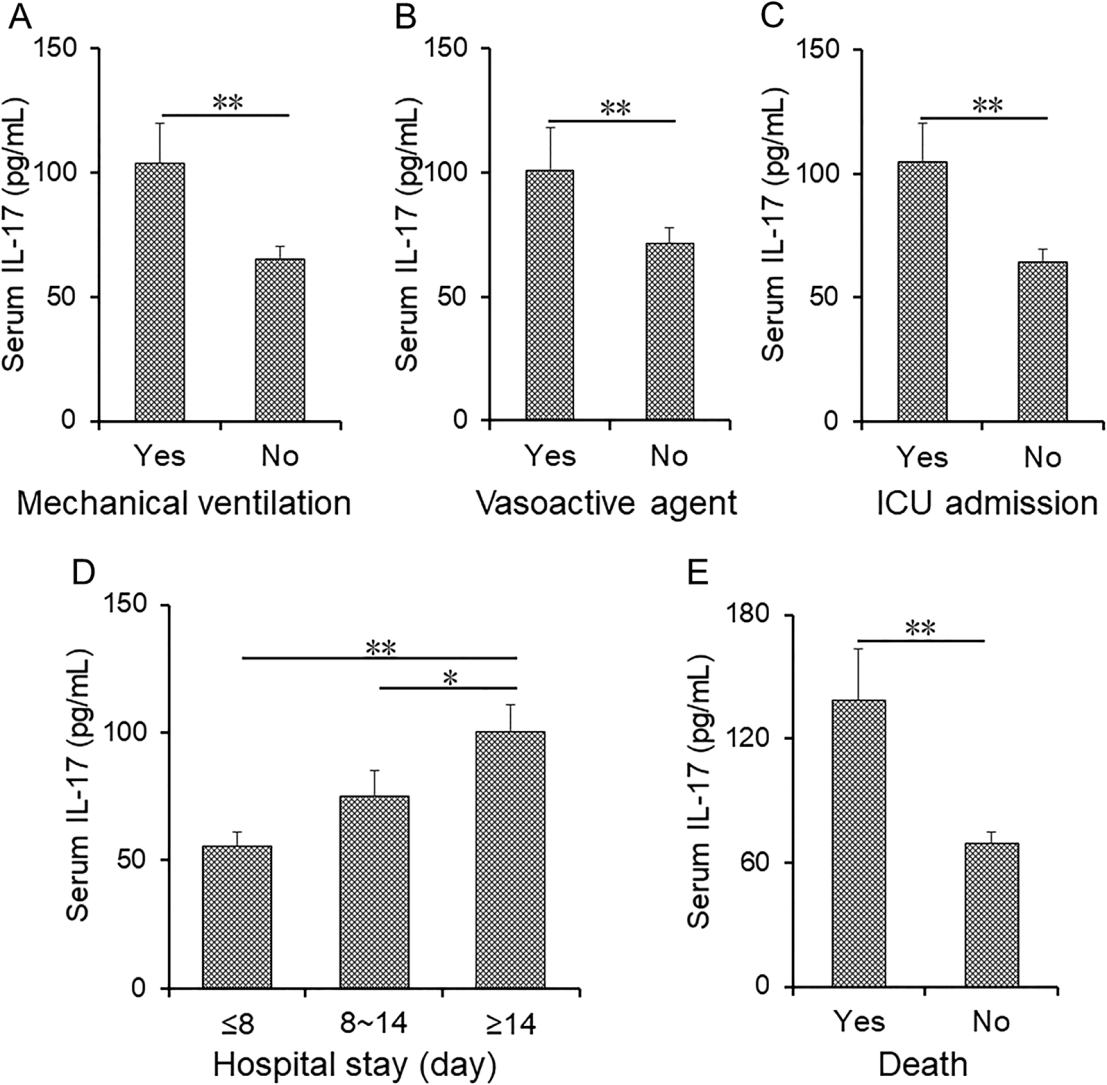
The levels of serum IL-17 in CAP patients with different prognostic outcomes. **A**–**E** The levels of serum IL-17 in CAP patients with different prognostic outcomes during hospitalizations. **A** Mechanical ventilation. **B** Vasoactive agent. **C** ICU admission. **D** Lengths of hospital stay. **E** Death. All data were expressed as mean ± SEM. *P < 0.05, **P < 0.01

Serum IL-17 was compared among CAP patients with different prognostic outcomes. As shown in Fig. 2A–C, the levels of serum IL-17 on admission were increased in patients with mechanical ventilation, vasoactive agent and ICU admission. Moreover, the levels of serum IL-17 on admission were elevated in patients with ≥ 14 hospital stays compared with patients with ≤ 8 hospital stays and from 8 to 14 hospital stays in CAP patients (Fig. 2D). In addition, serum IL-17 was further compared between alive and dead patients on admission. We found that serum IL-17 levels on admission were remarkably increased in the dead cases (Fig. 2E).

### The association between serum IL-17 and the prognosis in CAP patients

Univariate and multivariate logistical regression was performed to investigate the association between serum IL-17 and the prognosis of CAP patients. Univariable logistic regression identified that serum IL-17 on admission was positively associated with ICU admission (OR = 1.01; 95% CI 1.00, 1.02), mechanical ventilation (OR = 1.12; 95% CI 1.01, 1.42), death (OR = 1.05; 95% CI 1.00, 1.17) and ≥ 14 hospital stays (OR = 1.21; 95% CI 1.00, 1.51) (Table 4). For the sake of controlling some confounding factors, multivariate logistic regression was performed. The findings demonstrated that there were positive correlations between IL-37 with ICU admission (OR = 1.01; 95% CI 1.00, 1.01), mechanical ventilation (OR = 1.10; 95% CI 1.05, 1.37), death (OR = 1.05; 95% CI 1.00, 1.14) and ≥ 14 hospital stays (OR = 1.21; 95% CI 1.04, 1.57) among CAP patients (Table 4).

**Table 4:** Associations between serum IL-17 and the prognosis in CAP patients

	Univariable (95% CI)	*P*	Multivariable (95% CI)^a^	*P*
ICU admission	1.01 (1.00, 1.02)	< 0.01	1.01 (1.00, 1.00)	0.01
Mechanical ventilation	1.12 (1.01, 1.42)	< 0.01	1.10 (1.05, 1.37)	0.02
Vasoactive agent	1.00 (1.00, 1.01)	0.09	1.00 (1.00, 1.01)	0.18
Death	1.05 (1.00, 1.17)	< 0.01	1.05 (1.00, 1.14)	< 0.01
*Hospital stays*				
≤ 8	1	–	1	–
8–14	1.01 (1.00, 1.01)	0.08	1.00 (1.00, 1.01)	0.13
≥ 14	1.21 (1.00, 1.51)	0.03	1.21(1.04, 1.57)	0.01

^a^Adjusted for age and sex

### ROC curves and cut-off concentration of serum IL-17 for prognosis

**Fig. 3:**
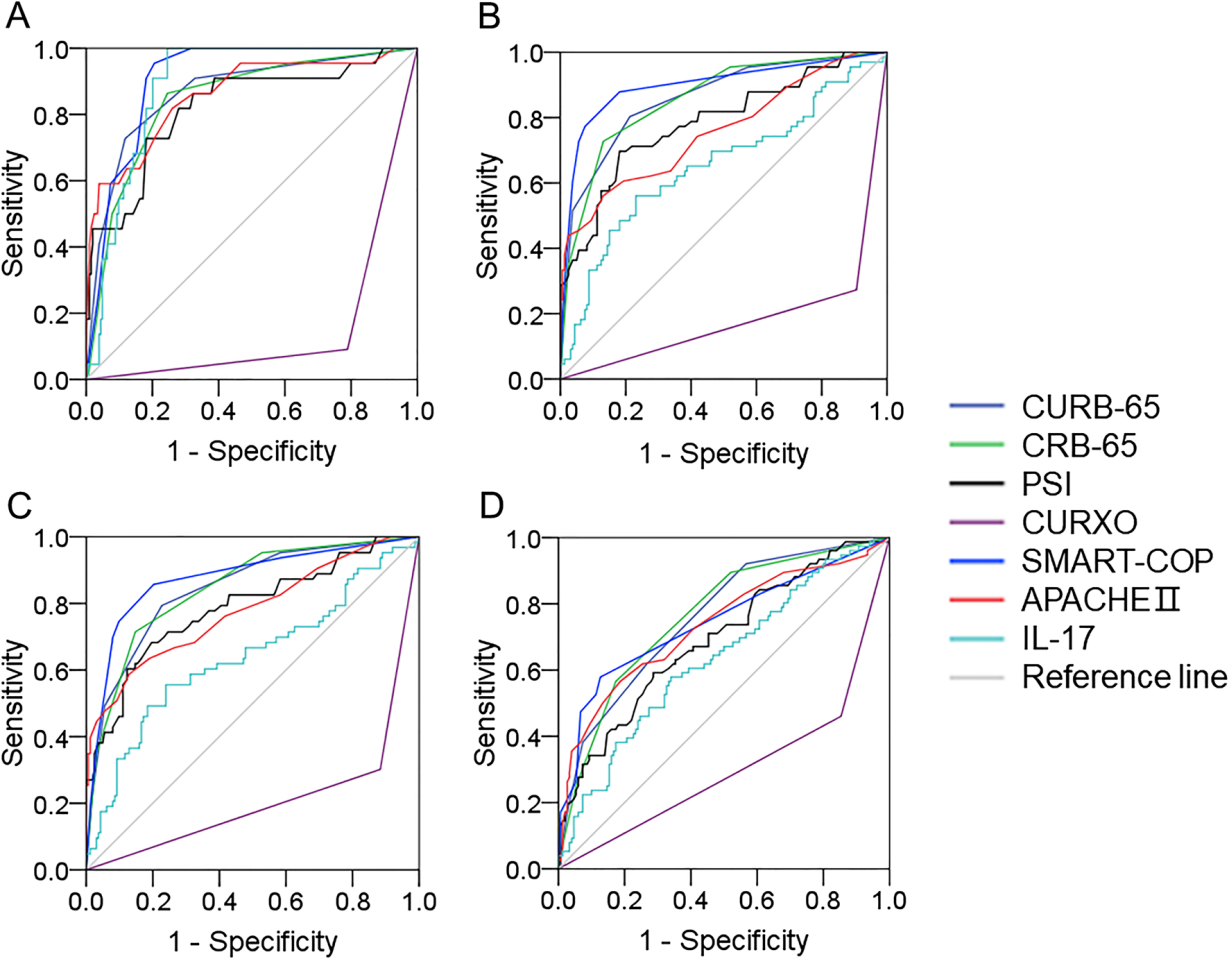
Receiver operating characteristic curves for prognosis in CAP patients. **A** ROC curve was used to evaluate the predictive values for death of different biomarkers among CAP patients. **B** ROC curve was used to evaluate the predictive values for ICU admission of different biomarkers among CAP patients. **C** ROC curve was used to evaluate the predictive values for mechanical ventilation of different biomarkers among CAP patients. **D** ROC curve was used to evaluate the predictive values for hospital stays of different biomarkers among CAP patients

The predictive capacities of IL-17 for prognostic outcomes were accessed using receiver operating characteristic (ROC) area under the curve (AUC) among CAP patients. As shown in Fig. 3A, the AUCs for death of serum IL-17, CURB-65, CRB-65, PSI, CURXO, SMART-COP and APACHE II were 0.89, 0.87, 0.85, 0.82, 0.15, 0.91 and 0.86. The cut-off concentration of IL-17 for death was 86.80 ng/mL in CAP patients. Moreover, the predictive powers for ICU admission were determined. As shown in Fig. 3B, the AUCs were as follows: IL-17, 0.65; CURB-65, 0.86; CRB-65, 0.86; PSI, 0.79; CURXO, 0.18; SMART-COP, 0.90; APACHE II, 0.76. The cut-off concentration of IL-17 for death was 84.92 ng/mL in CAP patients. As shown in Fig. 3C, the AUCs for mechanical ventilation were as follows: IL-17, 0.74; CURB-65, 0.84; CRB-65, 0.85; PSI, 0.79; CURXO, 0.21; SMART-COP, 0.87; APACHE II, 0.78. The cut-off concentration of IL-17 for mechanical ventilation was 84.92 ng/mL in CAP patients. Finally, the predicative capacities for ≥ 14 hospital stays were evaluated in CAP patients. Except lower predicative capacity of CURXO, there were similar predicative capacities for ≥ 14 hospital stays between serum IL-17 and CAP severity scores in CAP patients. The cut-off concentration of IL-17 for ≥ 14 hospital stays was 60.29 ng/mL (Fig. 3D).

## Discussion

This study strengthens the evidence of associations between serum IL-17 with the severity and prognosis among CAP patients. The research mainly found that: (1) Serum IL-17 on admission was gradually risen in parallel with CAP severity scores; (2) IL-17 on admission was closely correlated with many clinicopathological features among CAP patients; (3) IL-17 on admission was positively correlated with CAP severity scores in CAP patients; (4) Serum higher IL-17 on admission elevated the risk of ICU admission, mechanical ventilation, death and longer length of hospitalization among CAP patients during hospitalization.

IL-17, one of robust pro-inflammatory cytokines, which takes part in the airway inflammation, is a central factor in the excessive activation of the body’s defense system and the excessive inflammatory response [26]. The past studies demonstrated that IL-17 plays key roles in the progression of several lung diseases, including lung fibrosis, emphysema, acute lung injury and pulmonary hypertension [16–19]. Serum IL-17 is increased in patients with acute respiratory distress syndrome and participates in the occurrence of pneumonia [27, 28]. Nevertheless, the role of IL-17 in CAP was ill-defined so far. Therefore, we tested the levels of serum IL-17 among CAP patients with different severity scores. In the current research, we discovered that the level of IL-17 was gradually increased in parallel with the CAP severity scores among CAP patients. In addition, logistic and linear regression analysis indicated that IL-17 was positively associated with CAP severity scores among CAP patients. These findings indicated that IL-17 is positively correlated with the severity of CAP patients.

The previous studies from our laboratory have demonstrated that blood routine indices are obviously changed and the count of lymphocyte is dramatically reduced in COVID-19 patients [29–31]. Therefore, the associations of serum IL-17 and blood routine indices were evaluated among CAP patients in this cohort study. There were remarkable positive correlations between IL-17 with WBC and neutrophil, inverse correlations between IL-17 with lymphocyte and eosinophil in CAP patients. Moreover, our studies found that multiple organ dysfunction syndromes are always associated with the process of CAP [32–34]. IL-17 was positively correlated with uric acid, urea nitrogen, ALT, AST, CKMB and LDH among CAP patients. It is widely known that the occurrence of CAP is due to the invasion of pathogens, inflammatory cytokines and toxic metabolite [35]. Exorbitant inflammation evokes a serious of expanded cascade reaction, leading to systemic inflammation and multiple organ dysfunction syndromes [9, 36]. Other research groups have revealed that cytokine storm is observed in COVID-19 patients and inflammation suppression alleviated the severity of COVID-19 [37, 38]. In our present research, IL-17 was also shown to be prominently and positively related with inflammatory cytokines in CAP patients. These findings revealed that serum IL-17 may involve in the process of CAP and may be regarded as a biomarker for diagnosis and therapy for CAP patients.

Existing literatures have suggested that IL-17 is involved in the pathophysiology process and associated with the prognosis in pulmonary diseases. A research based on Chinese population found that IL-17 gene polymorphism is correlated with the risk and prognosis among patients with acute respiratory distress syndrome (ARDS) [39]. Several researches revealed that higher circulatory IL-17 is associated with the poor prognosis in patients with glioma and hepatocellular carcinoma [40, 41]. So, the association of IL-17 and the prognosis was estimated in CAP patients. Our results reflected that IL-17 on admission was elevated in CAP patients with ICU admission, mechanical ventilation, vasoactive agent, death and longer hospital stays during hospitalization. Logistic regression analysis revealed that serum higher IL-17 on admission was positively associated with the adverse prognostic outcomes among CAP patients. These results showed that the level of serum IL-17 on admission may predict the prognostic outcomes of CAP patients.

Limitations of the research were small sample size and single-center study, a larger sample size and multicenter study is needed to perform in the future. Additionally, due to the limitation of cohort study, only descriptive and correlational analyses were conducted. Further proofs based on basic experiments are needed to confirm this conclusion and mechanism. Besides, IL-17 was just measured in serum. In the next step, IL-17 would be detected in the bronchoalveolar lavage fluid and lung tissues of CAP patients.

## Conclusions

This research mainly demonstrated the correlations between serum IL-17 with the severity and prognostic outcomes of CAP patients based on a prospective cohort study. We found that IL-17 is gradually increased in parallel with the severity scores of CAP. Serum IL-17 is positively correlated with the severity and poor prognosis among CAP patients. The findings of this study indicated that IL-17 may be implicated in the pathophysiology processes of CAP. Therefore, it is likely that serum IL-17 may become an effective biomarker of diagnosis, prognosis and therapy for CAP patients in the future clinical works.

## Data Availability

The datasets used and analyzed during the current study are available from the corresponding author on reasonable request.

## References

[1] Mandell LA, Wunderink RG, Anzueto A (2007). Infectious Diseases Society of America/American Thoracic Society consensus guidelines on the management of community-acquired pneumonia in adults. Clin Infect Dis.

[2] Menéndez R, Torres A, Zalacaín R, Aspa J, Martín Villasclaras JJ, Borderías L, Benítez Moya JM, Ruiz-Manzano J, Rodríguez de Castro F, Blanquer J (2004). Risk factors of treatment failure in community acquired pneumonia: implications for disease outcome. Thorax.

[3] Heron M (2017). Deaths: leading causes for 2015. Natl Vital Stat Rep.

[4] Broulette J, Yu H, Pyenson B, Iwasaki K, Sato R (2013). The incidence rate and economic burden of community-acquired pneumonia in a working-age population. Am Health Drug Benefits.

[5] File TM, Marrie TJ (2010). Burden of community-acquired pneumonia in North American adults. Postgrad Med.

[6] Musher DM, Thorner AR (2014). Community-acquired pneumonia. N Engl J Med.

[7] Jackson ML, Nelson JC, Jackson LA (2009). Risk factors for community acquired pneumonia in immunocompetent seniors. J Am Geriatr Soc.

[8] Park H, Adeyemi AO, Rascati KL (2015). Direct medical costs and utilization of health care services to treat pneumonia in the United States: an analysis of the 2007–2011 Medical Expenditure Panel Survey. Clin Ther.

[9] Principi N, Esposito S (2017). Biomarkers in pediatric community acquired pneumonia. Int J Mol Sci.

[10] Aggarwal S, Ghilardi N, Xie MH, de Sauvage FJ, Gurney AL (2003). Interleukin-23 promotes a distinct CD4 T cell activation state characterized by the production of interleukin-17. J Biol Chem.

[11] Langrish CL, Chen Y, Blumenschein WM, Mattson J, Basham B, Sedgwick JD (2005). IL-23 drives a pathogenic T cell population that induces autoimmune inflammation. J Exp Med.

[12] Nograles KE, Zaba LC, Guttman-Yassky E, Fuentes-Duculan J, Suárez-Fariñas M, Cardinale I, Khatcherian A, Gonzalez J, Pierson KC, White TR (2008). Th17 cytokines interleukin (IL)-17 and IL-22 modulate distinct inflammatory and keratinocyte-response pathways. Br J Dermatol.

[13] Liang SC, Tan XY, Luxenberg DP, Karim R, Dunussi-Joannopoulos K, Collins M (2006). Interleukin (IL)-22 and IL-17 are coexpressed by Th17 cells and cooperatively enhance expression of antimicrobial peptides. J Exp Med.

[14] Kim EK, Kwon JE, Lee SY, Lee EJ, Kim DS, Moon SJ, Lee J, Kwok SK, Park SH, Cho ML (2017). IL-17-mediated mitochondrial dysfunction impairs apoptosis in rheumatoid arthritis synovial fibroblasts through activation of autophagy. Cell Death Dis.

[15] Baker KF, Isaacs JD (2018). Novel therapies for immune-mediated inflammatory diseases: What can we learn from their use in rheumatoid arthritis, spondyloarthritis, systemic lupus erythematosus, psoriasis, Crohn's disease and ulcerative colitis?. Ann Rheum Dis.

[16] Lei L, Zhao C, Qin F, He ZY, Wang X, Zhong XN (2016). Th17 cells and IL-17 promote the skin and lung inflammation and fibrosis process in a bleomycin-induced murine model of systemic sclerosis. Clin Exp Rheumatol.

[17] Mebratu YA, Tesfaigzi Y (2018). IL-17 plays a role in respiratory syncytial virus-induced lung inflammation and emphysema in elastase and LPS-injured mice. Am J Respir Cell Mol Biol.

[18] Gouda MM, Bhandary YP (2019). Acute lung injury: IL-17A-mediated inflammatory pathway and its regulation by curcumin. Inflammation.

[19] Wang L, Liu J, Wang W, Qi X, Wang Y, Tian B, Dai H, Wang J, Ning W, Yang T (2019). Targeting IL-17 attenuates hypoxia-induced pulmonary hypertension through downregulation of β-catenin. Thorax.

[20] Lowes MA, Suárez-Fariñas M, Krueger JG (2014). Immunology of psoriasis. Annu Rev Immunol.

[21] Boehncke WH, Schön MP (2015). Psoriasis. Lancet.

[22] Jiang X, Huang CM, Feng CM, Xu Z, Fu L, Wang XM (2021). Associations of serum S100A12 with severity and prognosis in patients with community-acquired pneumonia: a prospective cohort study. Front Immunol.

[23] Fang P, Zheng L, Cao P, Zhang C, Fei J, Xu Z, Feng CM, Zhao H, Lu YJ, Fu L (2020). Serum S100A8 as an early diagnostic biomarker in patients with community-acquired pneumonia. Arch Med Sci.

[24] Fu L, Zhao H, Xiang Y, Xiang HX, Hu B, Tan ZX, Lu X, Gao L, Wang B, Wang H (2021). Reactive oxygen species-evoked endoplasmic reticulum stress mediates 1-nitropyrene-induced epithelial-mesenchymal transition and pulmonary fibrosis. Environ Pollut.

[25] Fei J, Fu L, Cao W, Hu B, Zhao H, Li JB (2019). Low vitamin D status is associated with epithelial-mesenchymal transition in patients with chronic obstructive pulmonary disease. J Immunol.

[26] McAllister F, Henry A, Kreindler JL, Dubin PJ, Ulrich L, Steele C, Finder JD, Pilewski JM, Carreno BM, Goldman SJ (2005). Role of IL-17A, IL-17F, and the IL-17 receptor in regulating growth-related oncogene-alpha and granulocyte colony-stimulating factor in bronchial epithelium: implications for airway inflammation in cystic fibrosis. J Immunol.

[27] Li Q, Gu Y, Tu Q, Wang K, Gu X, Ren T (2016). Blockade of interleukin-17 restrains the development of acute lung injury. Scand J Immunol.

[28] Zhou J, Ren L, Chen D, Lin X, Huang S, Yin Y, Cao J (2017). IL-17B is elevated in patients with pneumonia and mediates IL-8 production in bronchial epithelial cells. Clin Immunol.

[29] Fu L, Fei J, Xu S, Xiang HX, Xiang Y, Hu B, Li MD, Liu FF, Li Y, Li XY (2020). Liver dysfunction and its association with the risk of death in COVID-19 patients: a prospective cohort study. J Clin Transl Hepatol.

[30] Fu L, Li XY, Fei J, Xiang Y, Xiang HX, Li MD, Liu FF, Li Y, Zhao H, Xu DX (2020). Myocardial injury at early stage and its association with the risk of death in COVID-19 patients: a hospital-based retrospective cohort study. Front Cardiovasc Med..

[31] Xiang HX, Fei J, Xiang Y, Xu Z, Zheng L, Li XY, Fu L, Zhao H (2021). Renal dysfunction and prognosis of COVID-19 patients: a hospital-based retrospective cohort study. BMC Infect Dis.

[32] Liu HY, Xiang HX, Xiang Y, Xu Z, Feng CM, Fei J, Fu L, Zhao H (2021). The associations of serum S100A9 with the severity and prognosis in patients with community-acquired pneumonia: a prospective cohort study. BMC Infect Dis.

[33] Wang JL, Chen X, Xu Y, Chen YX, Wang J, Liu YL, Song HT, Fei J, Zhao H, Fu L (2021). The associations of serum IL-37 with the severity and prognosis in patients with community-acquired pneumonia: a retrospective cohort study. Front Immunol.

[34] Zheng L, Fei J, Feng CM, Xu Z, Fu L, Zhao H (2021). Serum 8-iso-PGF2α predicts the severity and prognosis in patients with community-acquired pneumonia: a retrospective cohort study. Front Med.

[35] Choi SH, Hong SB, Ko GB, Lee Y, Park HJ, Park SY, Moon SM, Cho OH, Park KH, Chong YP (2012). Viral infection in patients with severe pneumonia requiring intensive care unit admission. Am J Respir Crit Care Med.

[36] Loughran AJ, Orihuela CJ, Tuomanen EI (2019). *Streptococcus pneumoniae*: invasion and inflammation. Microbiol Spectr.

[37] Kazama I (2020). Targeting lymphocyte Kv1.3-channels to suppress cytokine storm in severe COVID-19: Can it be a novel therapeutic strategy?. Drug Discov Ther..

[38] Zhang Y, Zhong Y, Pan L, Dong J (2020). Treat 2019 novel coronavirus (COVID-19) with IL-6 inhibitor: Are we already that far?. Drug Discov Ther.

[39] Xie M, Cheng B, Ding Y, Wang C, Chen J (2019). Correlations of IL-17 and NF-κB gene polymorphisms with susceptibility and prognosis in acute respiratory distress syndrome in a chinese population. Biosci Rep.

[40] Liao R, Sun J, Wu H, Yi Y, Wang JX, He HW, Cai XY, Zhou J, Cheng YF, Fan J (2013). High expression of IL-17 and IL-17RE associate with poor prognosis of hepatocellular carcinoma. J Exp Clin Cancer Res.

[41] Zhan X, Gao H, Sun W (2020). Correlations of IL-6, IL-8, IL-10, IL-17 and TNF-α with the pathological stage and prognosis of glioma patients. Minerva Med.

